# Preparation courses for medical clerkships and the final clinical internship in medical education – The Magdeburg Curriculum for Healthcare Competence

**DOI:** 10.3205/zma001039

**Published:** 2016-05-17

**Authors:** Anke Spura, Katrin Werwick, Annemarie Feißel, Marc Gottschalk, Kirstin Winkler-Stuck, Bernt-Peter Robra, Rüdiger C. Braun-Dullaeus, Philipp Stieger

**Affiliations:** 1 Otto-von-Guericke-Universität Magdeburg, Institut für Sozialmedizin und Gesundheitsökonomie, Magdeburg, Deutschland; 2 Otto-von-Guericke-Universität Magdeburg, Studiendekanat der Medizinischen Fakultät, Skillslab MAMBA, Magdeburg, Deutschland; 3 Universitätsklinikum Magdeburg, Universitätsklinik für Kardiologie und Angiologie, Magdeburg, Deutschland

**Keywords:** Medical studies, practice-oriented phases, internship "final clinical year" (PJ), clerkship, interprofessional collaboration, practical competence, preparatory seminar, skills workshop

## Abstract

**Background/Goals: **Supporting medical students entering their internships – the clinical clerkship and the internship “final clinical year” (Praktisches Jahr, PJ) – the seminars “Ready for Clerkship” and “Ready for PJ” were held for the first time in 2014 and continued successfully in 2015. These seminars are part of the “Magdeburg Curriculum for Healthcare Competence” (Magdeburger Curriculum zur Versorgungskompetenz, MCV). The concept comprises three main issues: “Understanding interdisciplinary clinical procedures”, “Interprofessional collaboration”, and “Individual cases and their reference to the system.” The aim of the seminar series is to prepare students as medical trainees for their role in the practice-oriented clinical clerkship and PJ, respectively.

**Methods: **Quality assurance evaluations and didactic research are integral parts of the seminars. In preparation for the “Ready for PJ” seminar a needs assessment was conducted. The seminars were rated by the participants using an anonymized questionnaire consisting of a 5-choice Likert scale (ranging from 1=fully agree to 5=fully disagree) and spaces for comments that was generated by the evaluation software Evasys.

**Results: **The results are presented for the preparatory seminars “Ready for Clerkship” and “Fit für PJ” held in 2014 and 2015. Overall, the students regarded the facultative courses as very good preparation for the clerkship as well as for the PJ. The three-dimensional main curricular concept of the MCV was recognized in the evaluation as a valuable educational approach. Interprofessional collaboration, taught by instructors focussing in teamwork between disciplines, was scored positively and highly valued.

**Conclusions: **The “Magdeburg Curriculum for Healthcare Competence” (MCV) integrates clerkship and PJ in a framing educational concept and allows students a better appreciation of their role in patient care and the tasks that they will face. The MCV concept can be utilized in other practice-oriented phases (nursing internship, bed-side teaching, block internships).

## Zusammenfassung

**Hintergrund/ Zielsetzung:** Zur Vorbereitung der Praxisphasen Famulatur und Praktisches Jahr (PJ) wurden im Jahr 2014 erstmalig die Seminare „Fit für Famulatur“ und „Fit für PJ“ durchgeführt und 2015 erfolgreich fortgesetzt. Das „Magdeburger Curriculum zur Versorgungskompetenz“ (MCV) bildet dafür den programmatischen Rahmen. Diese curriculare Leitidee umfasst drei Themenfelder „interdisziplinäre klinische Abläufe verstehen“, „interprofessionelle Zusammenarbeit“, „Einzelfall- und Systembezug“. Ziel ist es, die Studierenden für ihre Rolle in den Praxisphasen Famulatur und PJ zu stärken.

**Methodik: **Qualitätssichernde Evaluationen und Lehrforschung begleiten die Fit-für-Lehrveranstaltungen. Der Konzeption des „Fit für PJ“-Seminars ging außerdem eine Bedarfsanalyse zu möglichen Inhalten voraus. Die Fit-für-Seminarreihe wurde mit einem anonymisierten Fragebogen evaluiert, der aus einer 5-stufigen Likert-Skala („1=stimme voll zu“ bis „5=stimme nicht zu“) und Freitextfeldern bestand und mit der Evaluationssoftware Evasys generiert wurde.

**Ergebnisse: **Vorgestellt werden die Ergebnisse der Vorbereitungsseminare „Fit für Famulatur“ und „Fit für PJ“ 2014 und 2015. Insgesamt sahen die Studierenden das fakultative Lehrangebot als sehr gute Vorbereitung auf die anstehenden Famulaturen sowie das Praktische Jahr an. Die dreiteilige curriculare Leitidee wurde als sinnvoller didaktischer Ansatz in der Evaluation anerkannt. Interprofessionelle Zusammenarbeit wurde positiv bewertet, von einem interprofessionellen DozentInnen-Team umgesetzt und sehr geschätzt.

**Schlussfolgerung: **Das „Magdeburger Curriculum zur Versorgungskompetenz“ (MCV) integriert Famulatur und PJ in ein nachhaltiges didaktisches Gesamtkonzept und ermöglicht Studierenden, ihre Aufgaben/Rolle in der Patientenversorgung besser wahrzunehmen. Das MCV kann in weiteren Praxisphasen (Pflegepraktikum, UAK, Blockpraktika) eingesetzt werden.

## Authors

The authors A. Spura, K. Werwick, and P. Stieger contributed equally to the manuscript conception, draft, and revision and are therefore to be regarded equally as first authors.

## 1. Introduction

The aim of medical education is to produce “a physician who is educated in scientific and practice-oriented medicine who is able to practice the occupation in a self-reliant and independent manner and participate in further training and continuing education.” (ÄAppO §1, Abs. 1) [http://www.gesetze-im-internet.de/_appro_2002/BJNR240500002.html accessed on 13.08.15]. In spite of the successful incorporation of practical skills into medical education, there are still complex requirements at the beginning of a medical career, especially in the hospital setting, that are not part of the medical curriculum. The National Competence-Based Catalogue of learning objectives in Medicine (NKLM) from June 2015 and the recommendations of the Scientific Council (“Wissenschaftsrat der Bundesregierung”) for the Evaluation of Model Degree Programmes in Medicine are preparing a new direction for medical studies in Germany towards competence-oriented goals [9], [10], [11], [40], [http://www.nklm.de; accessed on 09.08.2015].

The practice-oriented phases of clerkship and the PJ play a crucial role in medical education. They promote the application of knowledge, skills and professional attitude in a concrete situation [21], [28]. They transfer theory into practice and socialize the specialized young physicians within the world of medicine. Interprofessional collaboration is becoming more important due to the increasing complexity of healthcare processes [7], [8], [40]. Therefore, medical education should focus early on a patient-oriented, multi-sector healthcare concept for prevention, healing, rehabilitation, and care [23], [38].

The seminar series “Ready for Clerkship” and “Ready for PJ” was first offered in Magdeburg in 2014 to prepare students for the practice-oriented phases of their medical education. In 2014-2015 the “Magdeburg Curriculum for Practice Competence” evolved into the “Magdeburg Curriculum for Healthcare Competence” (MCV), which combines both types of course offerings within one didactic framework. 

The students are prepared for their role in the practical phases as “novices on the periphery” [3] in the medical world and thus readied for their learning process. The aim of this article is to describe the concept of the MCV and report on initial experiences. The results of the evaluation of the preparatory seminars “Ready for Clerkship” and “Ready for PJ” for the years 2014 (pilot project) and 2015 are presented. 

## 2. Project Description - the MCV Concept

### 2.1. Dimensions of Competence 

**Figure 1:**
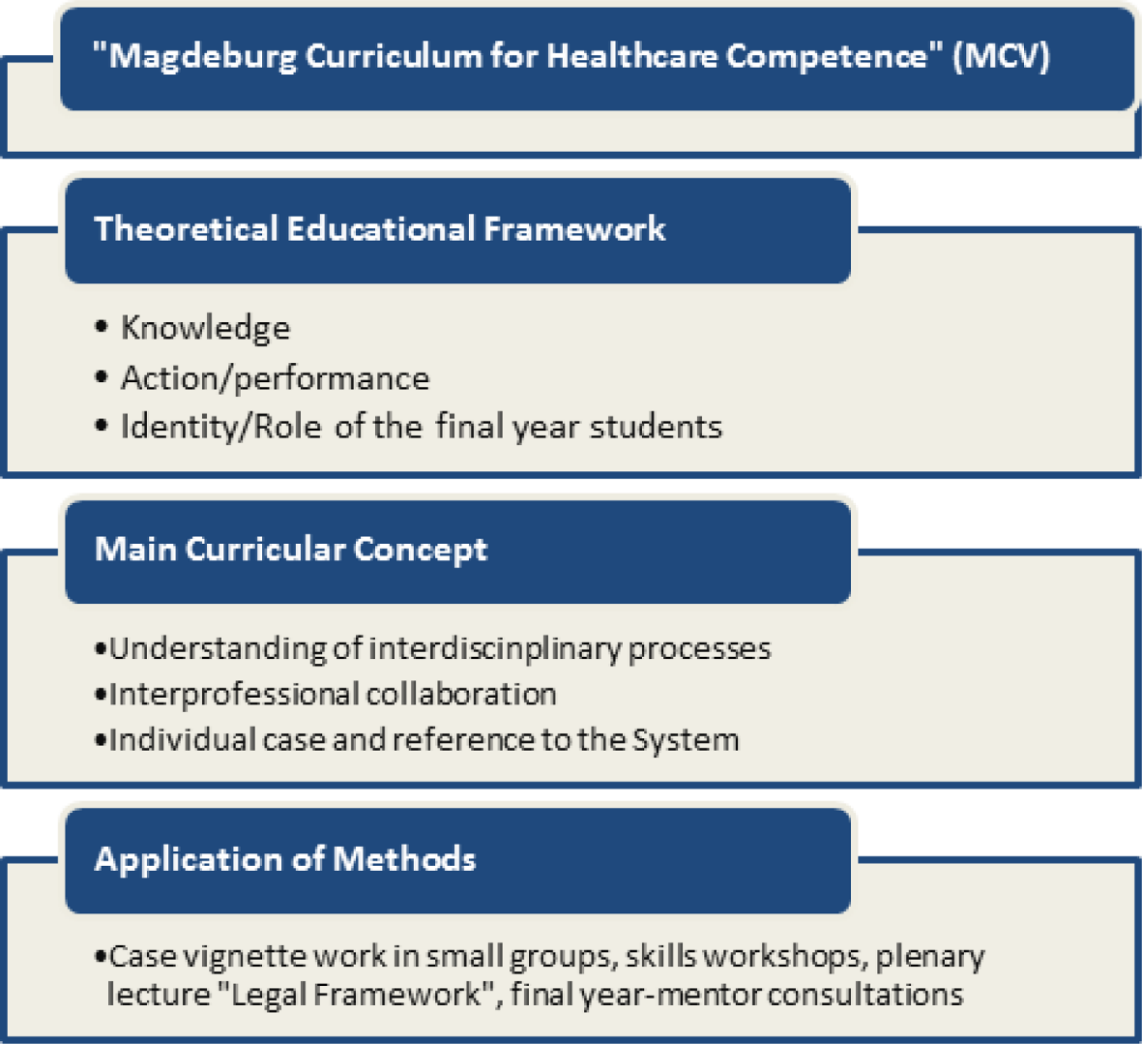
General scheme of the “Magdeburg Curriculum for Healthcare Competence” (MCV)

The MCV incorporates three occupational and future-oriented aspects of optimal medical care that serve as a main curricular concept: understanding of interdisciplinary processes, interprofessional cooperation, and the application of individual cases with reference to the system as a whole (see Figure 1 (Fig. 1)) [33]. The aim of a healthcare competence-oriented medical education are familiarization “with the medical care of patients” (ÄAppO 2002, §7 Abs. 1) during the clerkship and the integration within healthcare processes through the guided accomplishment of medical duties during the PJ (ÄAppO 2002, §3 Abs. 4) [http://www.gesetze-im-internet.de/_appro_2002/BJNR240500002.html, accessed on 13.08.2015].

Since courses for students to prepare for their clerkship or PJ have also been conducted successfully at other locations, they are primarily directed at the acquisition of practical skills [1], [4], [6], [13], [15], [19], [32], [34]. Beyond the mere transfer of skills, the MCV emphasizes the responsive, contextual aspects that focus on the beginning of the medical career. The approach is based on the idea of medical education being an educational-biographical process [5]; stages of the physician’s professional status [38] are strengthened by clinical implementation and taking on clinical responsibility. This process goes beyond the transfer of knowledge and competence or the thought processes involved in socialization (20). Therefore, the application of the MCV methods is based on the three dimensions of competence “Knowledge”, “Action/Performance”, and “Identity/Role” (see Figure 1 (Fig. 1)), which are part of classical [27], [29] and contemporary international discourses on competence [14], [http://www.oecd.org/edu/skills-beyond-school/41529505.pdf accessed on 13.08.2015]. 

#### Knowledge

Knowledge as an integral part of medical competence includes an understanding of bio-psycho-socio relationships between the origin and treatment of diseases and health as well as knowledge about the organization of clinical therapy processes, healthcare practices used in different sectors, and the bureaucratic and legal frameworks within the field of medicine. 

##### Action/Performance

In addition to manual medical skills, the orientation of the MCV is directed toward the performance level [11] of professional demeanour, such that knowledge, professional demeanour, skills, and identity are realized in concrete medical practice applications. Informal and implicit logic [26], which becomes effective during the execution of medical tasks, backs up transferred skills.

##### Identity/Role

Students must define their position as clerk or PJ student at the latest at the outset of their internship on a ward or within a practice team so that they can incorporate their role and status into the workplace atmosphere accordingly. In doing this they should not lose sight of their chance to learn from everyday practice. The reference to the identity [17] of the students completing their clerkship or PJ means the ability to reflect on their own professional role that they take on in the practice-oriented phase, i.e. to become self reliant. 

#### 2.2. Main Curricular Concepts

Participants of the “Ready for” seminars framed by the MCV should be able to

1. understand interdisciplinary clinical processes,
2. work interprofessionally, and
3. view an isolated medical case as part of the entire context of the healthcare system.

The trinity of “understanding interdisciplinary clinical processes”, “interprofessional cooperation”, and the “individual case with reference to the system” forms the core programme of the MCV and the seminar series “Ready for Clerkship” and “Ready for PJ”. It is focused on the beginning of the medical career, and during the course of the seminars – based on a thematic series of case vignettes – it is transfered from a theoretical into a practical context, i.e. into a healthcare concept. Knowledge, actions, and the role of the medical clerk or PJ/intern are continually reflected in this process. Thus, the key points of the MCV mentioned above – as related to the role of the NKLM in medical competence [12], which is based partly on the role aspects of the CanMeds [18], [35] – constitute the basis of competence of a clerkship/PJ, as healthcare competence. To transfer this open curriculum as learning objectives is up to the instructor of the individual modules.

##### 2.2.1. Understanding Interdisciplinary Clinical Procedures

First main topic of the MCV programme imparts knowledge transfer for treatments of complex diseases, for disciplinary responsibilities for the implementation of diagnosis and therapy as well as for documentation and for intersectoral communication in the health system.

An ageing population, an increase in multimorbidity, and the reality of work place conditions in hospitals require more interdisciplinary collaboration in future medical care. It is therefore essential to sensitize students for interdisciplinary collaborations and various professional healthcare strategies.

##### 2.2.2. Interprofessional Cooperation 

Working relationships at a clinic or in a practice are determined by the collaboration of various professional groups, e.g. nursing, social work, emergency services, and their cooperation partners. This topic of the MCV focusses on learning the specific professional competencies to understand the division of labour of organized healthcare processes, and to be part of these interprofessional organized health care practices. For this reason, there are representatives of various professions as lecturers.

##### 2.2.3. Individual Case and System-Reference

The definition of an individual clinical case and its allocation in treatment paths requires the consideration of disease specific, familiar, occupational, social, and biographical contexts of the patient (the patient’s life-world). Medical care standards based on medical knowledge [36] within the binary “health-desease” code [22] need to be transfered into individualized patient-oriented health care. Here, aspects of ward management during periods of understaffing [37] or healthcare structures that bridge different sectors come into play (relation to the medical system). General principles of medical care are transformed into a clinical case vignette. 

The practical patient-oriented and the system-oriented learning objectives, which are both required by the the medical approbation rules (“insight and integration into patient care”, “healthcare of the population” (ÄAppO 2002, §1 Abs. 1) [http://www.gesetze-im-internet.de/_appro_2002/BJNR240500002.html, accessed on 13.08.2015]), are taken into account.

## 3. Implementation

The didactic approach is realized by case vignettes and accompanying small-group skills-refresher workshops. In addition, various workshops, for instance dealing with the legal ramifications of the practical phases and PJ-mentor consultations, are held to help in defining the role of the student interns (see Figure 1 (Fig. 1)). 

### 3.1. Participants

The seminar series is directed at students of the Magdeburg medical school who want to carry out their clerkship/PJ at teaching hospitals or registered “academic teaching medical practices.” The didactic program of the MCV connects for the first time two practical internships of medical education.

#### 3.2. Seminar Structure

The seminars series for clerkship and PJ have a similar modular structure referring to contemporary topics of workplace reality of physicians:

- Ward management
- Emergency situations
- Localization within the medical care system
- Teamwork capability
- Interface for inpatient and outpatient care
- Handling specialty-specific burdens

The focus in terms of an exemplary training plan is on the integration of a typical medical case within a systematic clinical healthcare process. Under the supervision of medical and nursing instructors, students should become familiar with healthcare concepts and ward procedures and should be able to make the connection between the processes and structures behind these concepts and their own theoretical knowledge base. In this way, general healthcare principles can be worked out together. Based on a case vignette, students learn supervised by medical and nursing instructors work on the themes of ward rounds and communication during shift changes of the medical staff. Special workshops, led by nursing instructors, deal with the role of patient management, introduce to the handling with medical equipment (e.g. the use of an infusion pump of patient monitoring equipment), and introduce to work routines in the OR; this serves to illustrate various interprofessional role models and expectations. Case vignette based workshops are complemented by respective skills workshops, which comprise a further, defined part of the module. These workshops focus on necessary skills and requirements for the students’ activities on the ward and are thematically structured and tuned to the medical case being discussed. The core programme is supplemented by various offerings by experts (specialized seminars such as “Legal Framework of the Clerkship/PJ”, “Meet the Expert”, “Market of Opportunities”, “Student-Mentor Consultations”) that are adapted to the various stages of practice and will contribute to strengthening the role of the medical student in a stationary care environment. 

##### 3.2.1 “Ready for Clerkship”

This seminar is focused on structuring the teaching/learning processes in the clerkship. The aim of the clerkship is to provide insight into patient care (see ÄAppO §7) [http://www.gesetze-im-internet.de/_appro_2002/BJNR240500002.html accessed on 13.08.2015]. The seminar is offered once a year as an elective two-day preparatory course (see Table 1 for the organization (Tab. 1)) and designed primarily for medical students before their first clerkship. There are currently 55 students (from approx. 200 potential participants) enrolled who are instructed in small groups of 8-10 (see Table 2 (Tab. 2)). 

The first part of each module concentrates on thematically organized basic aspects of a medical case description in clinical care starting as an internal case and then transferred into a surgical one. In the concluding segment of the respective module, basic concepts of clinical skills needed for patient care on the ward are taught. The aim of the course is the consolidation of the role of the medical clerk as a participating observer of clinical practice with the aim of strengthening medical skills.

##### 3.2.2. “Ready for PJ”

The seminar series “Ready for PJ” is offered as a one-week elective course prior to the beginning of the final clinical internship PJ. The course has a capacity of 60 students (from approx. 200 potential participants). The teaching method is primarily based on small-group sessions. In the morning the learning is problem-oriented organized around a case vignette that changes thematically as the week progresses. Students should reflect their own role in the "arc of work" [33] of health care routines on the ward. Accompanying plenary lectures deepen the knowledge or summarize the daily exercises (“Cardiac Patients in the Medical Rounds”, “Imaging”, “Wound Assessment”, “Writing a Medical Report”). The second part in the afternoon comprises thematically organized parallel skill-refresher workshops (see Table 3 (Tab. 3)). 

The aim of this course is the improvement of clinical competencies as related to the tasks and the realities of the workplace of student interns in a stationary care environment.

## 4. Evaluation and Accompanying Educational Development

All courses were accompanied by quality evaluations and educational research (see Section 5, Results of Evaluation). This was carried out in part within the framework of a qualitative, guideline-based dissertation research on the clerkship experience with “Ready for Clerkship” participants who had successfully completed their first clerkship after both cycles in 2014 and 2015. 

In addition, an in-depth standardized questionnaire, still being evaluated, was administered to “Ready for Clerkship” students after their first clerkship in 2015 in which they provided information regarding their expectations in internal medicine and surgical specialties. 

An analysis of possible course content (needs assessment) was made in advance of the “Ready for Clerkship” evaluation that involved the participating instructors and internship mentors (N=5), former interns (N=26), and current student participants (N=47, 2014 and N=28, 2015). In the summer of 2015 a qualitative, structured email survey of the participants was performed [2], [16], [24] with respect to their current internship experience following their participation in the pilot project in 2014.

The seminar evaluation was accomplished with an anonymized questionnaire that was generated with the evaluation software Evasys. The questions consisted of a 5-choice Likert scale (“1=fully agree” to “5=fully disagree”) and fields for comments. The students rated course organization, implementation of the main concept, and all course segments and instructors as well as subjective learning progress. The data obtained were evaluated with statistical software SPSS (Version 21).

## 5. Results

### 5.1. Evaluation of the Seminar “Ready for Clerkship” 2014, 2015

Approximately three quarters (77 percent) of the course participants from 2014 and 2015 took part in the evaluation. The participants as a whole viewed the elective course as a very good preparation for the clerkships. They gave an average rating of 1.64±0.64 for 2014 and 1.48±0.68 for 2015. 

**Figure 2:**
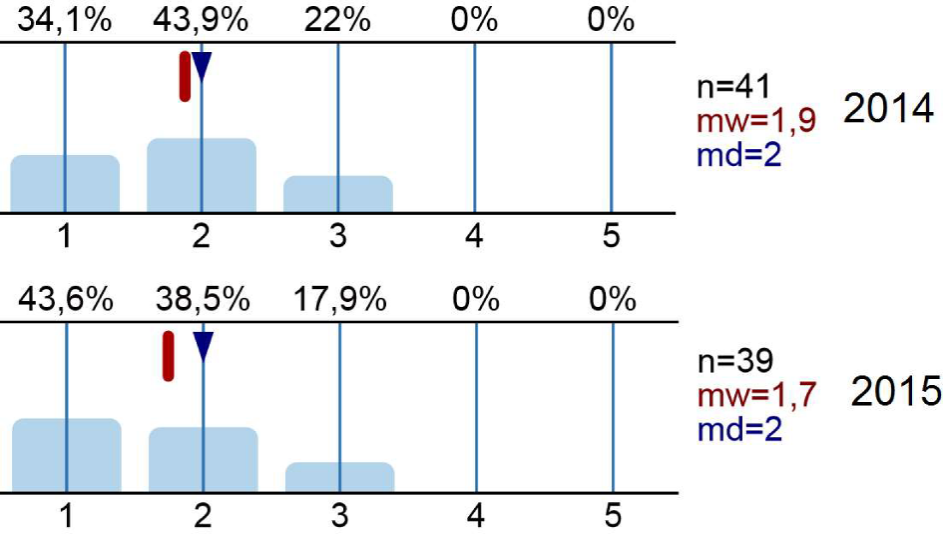
“Ready for Clerkship” 2014, 2015 – “After participating in the seminar I have a better idea of how I can integrate myself into the team on the ward.” 1=“Fully agree” to 5=“Fully disagree”

The majority of the students rated the seminar organization as very good for both years (average rating=1.4±0.49). The level of satisfaction with the extent of the training in small groups, in case work, and in mastering of skills was increased from 2014 to 2015: in 2014 half of the participants mentioned the need for improvement, and thus in 2015 the concept was adjusted to accommodate the results of the 2014 evaluation and the training time was modified. In parallel, most of the students agreed (average rating=1.4 for 2015; 1.7 for 2014) that the knowledge gained could be applied in the internship. After the seminar series, most of the students had a better idea of how they could play a role in the team on the wards than beforehand (see Figure 2 (Fig. 2)).

Regarding the comments of the free-text fields the students of both years gave support for the training methods using vignette-based casework in small groups. According to the students, the seminar series provided new insights into daily clinical practice. The high degree of motivation of the instructors was praised. In particular, the teaching provided by the instructors from the nursing field was positively rated, and suggestions were made for thematic as well as staff enhancements. Furthermore, more time for practicing skills and for the themes “Taking Patient Histories” and “Communication on the Ward” was recommended. 

#### 5.2. Evaluation of the Seminar “Ready for PJ” 2014, 2015

A total of 48 students were enrolled in the first preparation course in 2014, and fewer participated in the evaluation (n=28, with n=20 female) than in the following year, with a total of n=60 students enrolled in 2015 (n=42 participated in the evaluation, n=29 female). 

The evaluation by the medical students demonstrated that the “Ready for PJ” course was an excellent preparation for the internship with an average rating of 1.65 (2014) and 1.7 (2015). Participants regarded the course as being relevant for the ensuing tasks during the internship and in general would recommend it to other students. In 2014 93percent agreed that the discussions were constructive and that the knowledge acquired could be applied during the internship. This was also evaluated positively in 2015 with 76 percent agreeing. 

The analysis of needs assessment carried out prior to the course regarding preparation for the PJ and subjective evaluation of their own abilities showed only “satisfactory” results for the students; the results of the evaluation of the seminar documented a marked improvement in self-evaluation (see Table 4 (Tab. 4)).

The issue “Interprofessional Collaboration” was in particular successfully implemented: 89.3 percent of the survey participants agreed or fully agreed that they could better appreciate the perspective of non-medical professional groups than before the “Ready for PJ” course. The future internship participants also indicated that they had a better idea of how they could integrate themselves into a team on the wards. After participating in “Ready for PJ” the students reflected more on the consequences of their decisions for happenings on the wards than before the course (82.1 percent agreed fully); clinical decision-making processes could be better understood by the majority (92.9 percent). 

**Figure 3:**
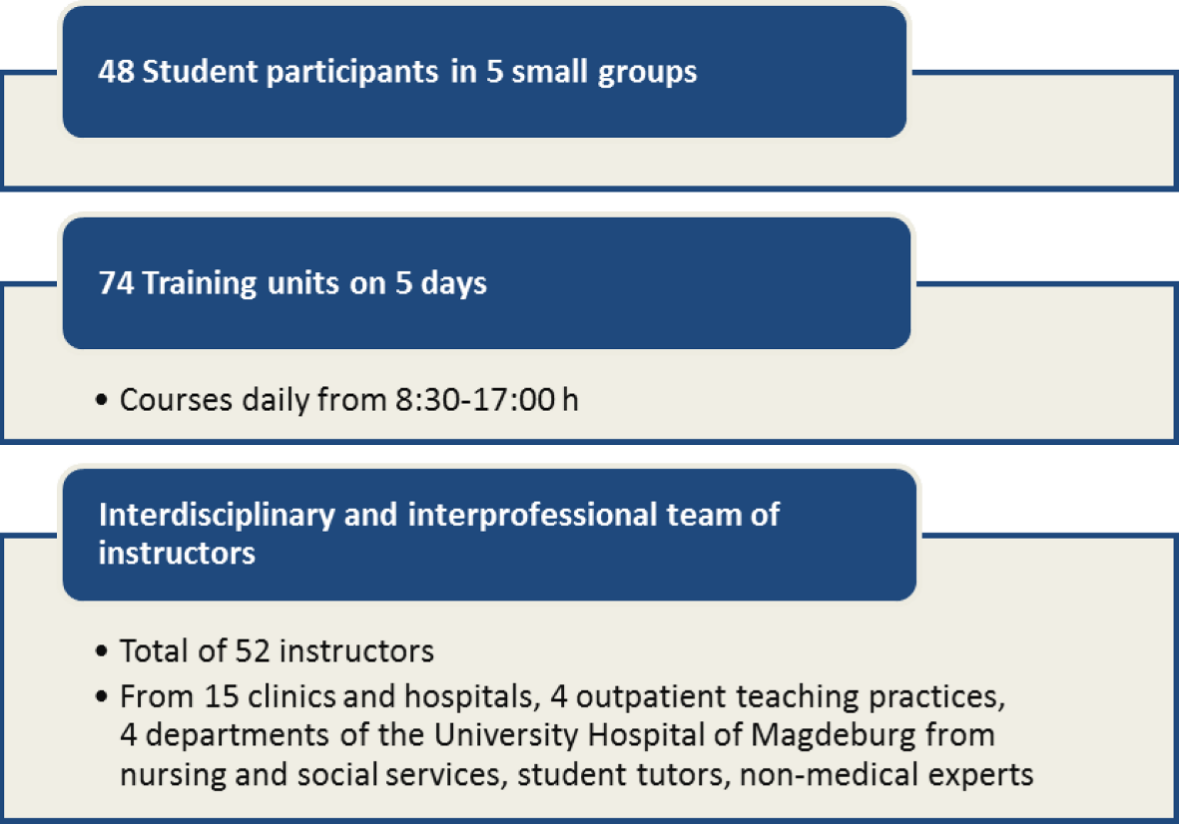
Pilot project "Ready for PJ" 2014

Furthermore, 93 percent of the seminar participants valued the learning atmosphere with constructive discussions as being very pleasant. The evaluation of the course revealed a desire for more time for practice, and only one third found the practice time to be adequate. Taking this desire into consideration could only be realized by reducing or changing several of the modules, as the weekly planning is already very compact (see Table 1 (Tab. 1) and Figure 3 (Fig. 3)). In their comments more than 30 percent praised the relevance of the material to clinical practice and for the strengthening of practical skills.

All modules in the weekly plan (see Table 1 (Tab. 1)) were rated on average as “very good” or “good” (see Table 5 (Tab. 5)). Between the individual teaching groups there were slight differences in the module and instructor ratings.

The comments show particularly positive results for the documentation training for “Consultation Requirement” and “Medical Regulations” as well as for the issue “Medical Rounds”. The vignette work also found appeal. Several participants reported increased motivation because fears were dispelled and the role of the interns was better appreciated from the viewpoint of the instructors. 

## 6. Discussion and Perspectives

The seminar series reflects the current discourse concerning the improvement of medical school internship. It helps to establish the clerkship and final clinical internship PJ as a holistic didactic concept that complements the already successful courses directed at improving patient- and care-oriented practical skills (bedside training, clinical block internships, skills workshops). 

Our analysis demonstrates that the MCV concept can be implemented. It has a positive resonance, and is perceived by students and instructors alike as being necessary. At the current stage, the analysis does not allow an empirical conclusion regarding for example the experiences in clinical (team)work of all participants; this critical aspect will be addressed in further educational research projects. The sustainability of the training initiative will become apparent after several additional cycles and parallel research. 

Beyond knowledge- and practice-oriented training initiatives, students could be strengthened in their medical trainee role in clerkship and PJ. Internship trainees can hardly be expected to take over the supervisory responsibilities for diagnostics, treatment planning, and therapy from their medical instructors who are working under a heavy caseload in everyday ward practice [29]. The “Ready for-“ seminar series focusses these current issues and supports an unencumbered learning atmosphere purposefully removed from everyday ward practice such as that offered in the protected space of a skills workshop. This provides a benefit for both students and instructors just before the beginning of the practice-oriented phase. 

All three issues of the curriculum of the MCV were recognized as being important didactic approaches. The focus on interprofessional collaborations is not only anchored in the MCV as a main cornerstone; the seminar participants also gave the nursing staff high recognition as competent and relevant instructors. The MCV can be applied to further practice-oriented phases (nursing internship, bed-side teaching, block internships).

## Acknowledgements

The authors wish to thank especially their consulting and advisory partners, in particular

- the team of interdisciplinary and interprofessional instructors of this programme,
- the Director of Nursing at the University hospital , Magdeburg, Germany, Dipl. Nurse D. Halangk,
- the Director of the University Clinic for Trauma Surgery, Magdeburg, Germany, Prof. Dr. F. Walcher,
- the Head of Geriatrics and Palliative Care at the Pfeiffersche Stiftung Clinic, Magdeburg, Germany, Dr. G. Heusinger von Waldegg,
- the Dean of the Medical Faculty, Magdeburg, Germany, Prof. Dr. H.-J. Rothkötter,
- the Vicedean for Education (Studiendekan) of the Medical Faculty, Magdeburg, Germany, Prof. Dr. C. H. Lohmann,
- the medical students

for their comments, suggestions, and support. We thank the medical faculty for the financial support for this training project. 

## Competing interests

The authors declare that they have no competing interests.

## Figures and Tables

**Table 1:**
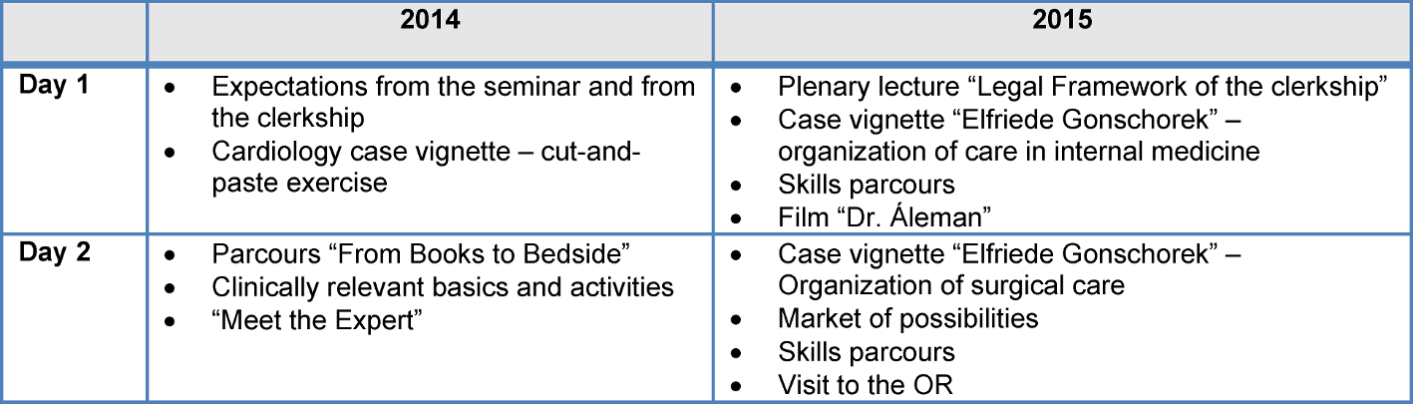
Course organization of "Ready for the Clerkship" 2014 (pilot project) and 2015

**Table 2:**
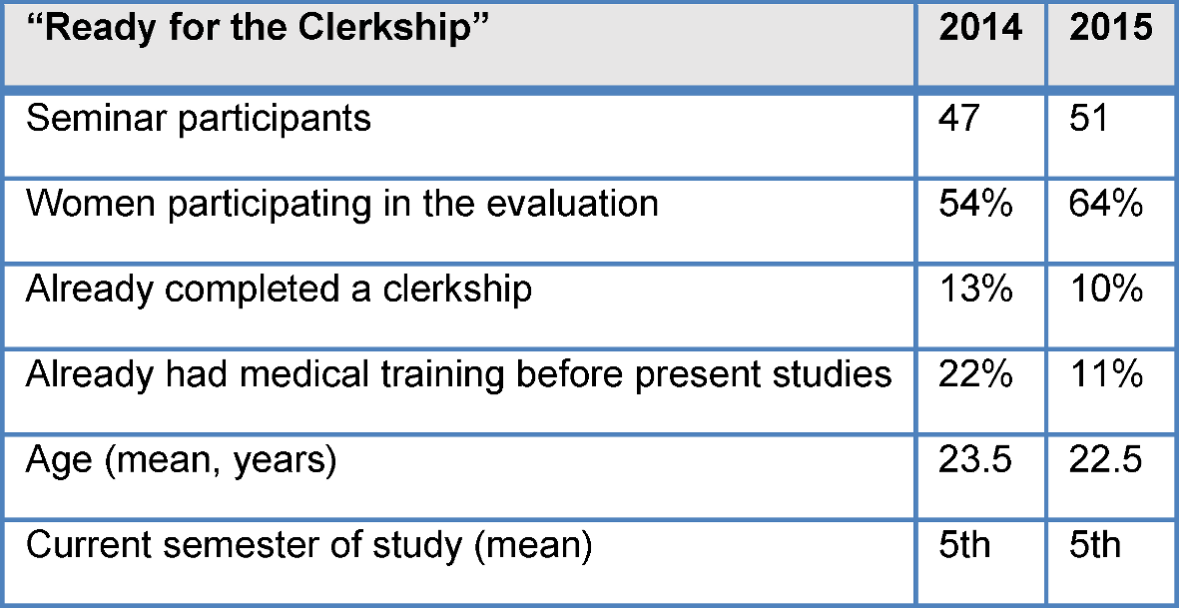
Participants in “Ready for Clerkship” 2014 and 2015

**Table 3:**
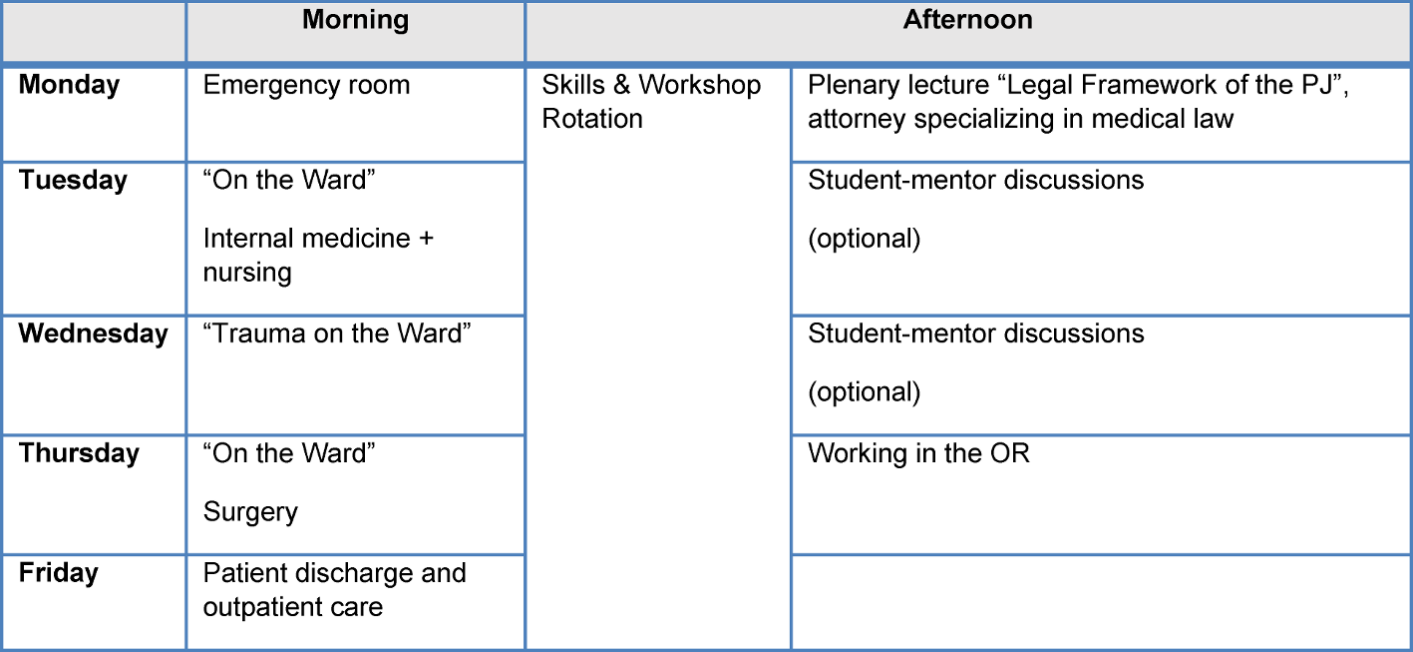
Course organization “Ready for PJ” 2014 (pilot project)

**Table 4:**
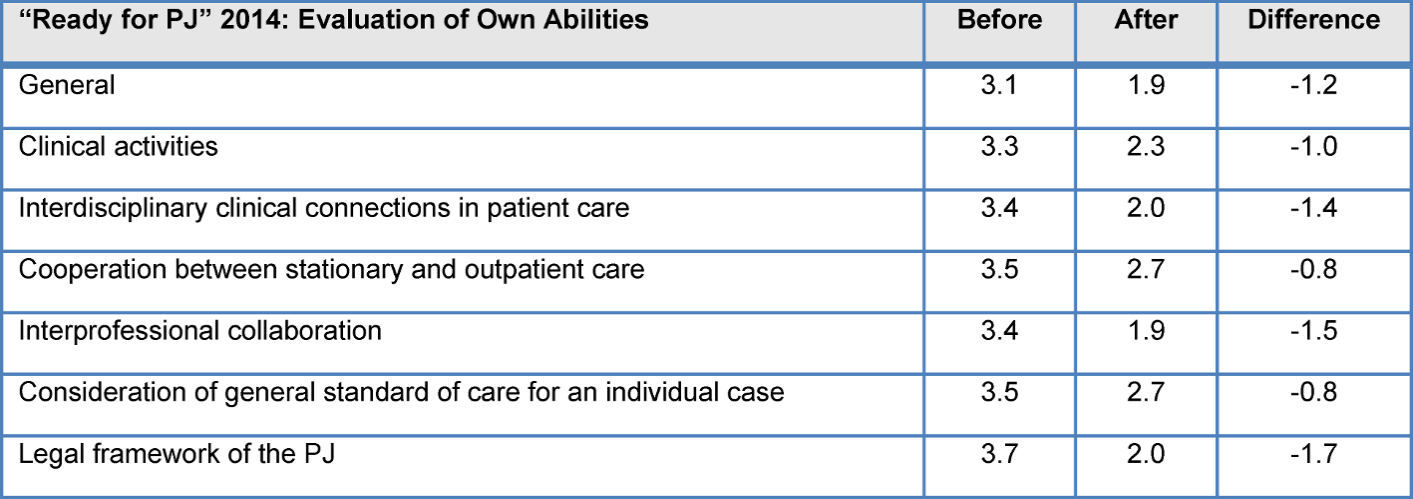
Comparison of the results of the needs analysis– Evaluation of selected items (“How well prepared for the PJ do you feel beforehand?” vs. “How well prepared for the PJ do you feel after participating the “Ready for PJ” course?”) (1=“Fully agree/very good” to 5=“Fully disagree/very bad”) 2014

**Table 5:**
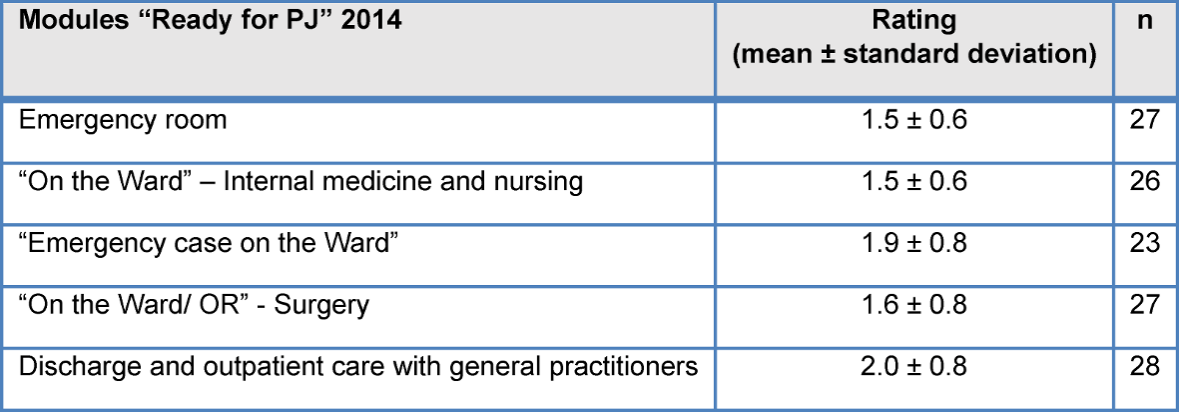
Module evaluation "Ready for PJ" 2014, (1=“very good” to 5=“very bad”)
